# Stoma Rate and Oncological Outcomes of Primary TaTME vs Completion TaTME in Patients With Early-Stage Rectal Cancer

**DOI:** 10.1097/DCR.0000000000003794

**Published:** 2025-05-05

**Authors:** Annabel S. van Lieshout, Laura R. Moolenaar, Floor F.A.C. Tobben, Stefan E. van Oostendorp, Lisanne J.H. Smits, Jeroen C. Hol, Eric H.J. Belgers, Eric J.T. Belt, Steven J. Oosterling, Colin Sietses, Pascal G. Doornebosch, Roel Hompes, Jurriaan B. Tuynman

**Affiliations:** 1 Department of Surgery, Amsterdam UMC, Vrije Universiteit Amsterdam, Amsterdam, the Netherlands; 2 Cancer Center Amsterdam, Treatment and Quality of Life, Amsterdam, the Netherlands; 3 Cancer Center Amsterdam, Imaging and Biomarkers, Amsterdam, the Netherlands; 4 Department of Surgery, Zuyderland Medical Center, Heerlen, the Netherlands; 5 Department of Surgery, Department of Surgery, Albert Schweitzer Hospital, Dordrecht, the Netherlands; 6 Department of Surgery, Spaarne Gasthuis, Hoofddorp, the Netherlands; 7 Department of Surgery, Gelderse Vallei Hospital, Ede, the Netherlands; 8 Department of Surgery, IJsselland Hospital, Capelle a/d Ijssel, the Netherlands

**Keywords:** Completion surgery, Early rectal cancer, Local excision, Stoma, Transanal total mesorectal excision

## Abstract

**BACKGROUND:**

Local excision as a first step in the treatment of early rectal cancer has gained interest. However, in the presence of histopathological risk factors, (inter)national guidelines recommend completion total mesorectal excision. Although oncologically safe, completion total mesorectal excision is associated with an increased end colostomy rate compared to primary total mesorectal excision, especially in distal lesions. Transanal total mesorectal excision may facilitate lower anastomoses, potentially reducing end colostomy rates.

**OBJECTIVE:**

To compare the end colostomy rate and oncological outcomes of primary transanal total mesorectal excision with local excision followed by completion transanal total mesorectal excision in patients with cT1-2N0M0 rectal cancer.

**DESIGN:**

Data were prospectively collected and retrospectively analyzed.

**SETTINGS:**

This study was conducted in 6 Dutch high-volume centers experienced in transanal total mesorectal excision.

**PATIENTS:**

All patients with cT1-2N0M0 rectal cancer who underwent primary transanal total mesorectal excision or local excision followed by completion transanal total mesorectal excision between 2012 and 2022.

**MAIN OUTCOME MEASURES:**

The primary outcome was end colostomy rate. Secondary outcomes included anastomotic leakage, involvement of the circumferential resection margin, specimen quality, local recurrence, distant metastases, overall survival, and disease-free survival.

**RESULTS:**

A total of 150 patients were included with a median follow-up of 32 and 23 months for primary transanal total mesorectal excision and local excision followed by completion transanal total mesorectal excision, respectively. The end colostomy rate was significantly lower in the local excision followed by completion transanal total mesorectal excision group (21%) compared to the primary transanal total mesorectal excision group (42%, *p* = 0.022). More anastomotic leakages occurred in the local excision followed by completion transanal total mesorectal excision group (33% vs 18%, *p* = 0.064). No differences were observed in circumferential resection margin involvement and specimen quality. Two-year local recurrence rates were 4% for primary transanal total mesorectal excision and 3% for local excision followed by completion transanal total mesorectal excision (*p* = 0.343), whereas distant metastases occurred in 8% and 10% (*p* = 0.424), respectively. There were no significant differences in 2-year overall survival (88% vs 97%, *p* = 0.101) and 2-year disease-free survival (82% vs 90%, *p* = 0.463) between groups.

**LIMITATIONS:**

The small sample size, which precluded correction for group differences, and selection bias.

**CONCLUSIONS:**

This study demonstrated that local excision followed by completion transanal total mesorectal excision for cT1-2N0 rectal cancer neither increased the end colostomy rate nor compromised oncological outcomes compared to primary transanal total mesorectal excision in experienced centers. See **Video Abstract**.

**TASA DE ESTOMAS Y RESULTADOS ONCOLÓGICOS DE LA TATME PRIMARIA FRENTE A LA TATME DE COMPLEMENTO EN PACIENTES CON CÁNCER RECTAL EN ESTADIO TEMPRANO:**

**ANTECEDENTES:**

La escisión local como primer paso en el tratamiento del cáncer rectal en estadio temprano ha despertado interés. Sin embargo, en presencia de factores de riesgo histopatológicos, las directrices internacionales recomiendan la escisión mesorrectal total completa. Aunque es segura desde el punto de vista oncológico, la escisión mesorrectal total de complemento se asocia con una mayor tasa de colostomía terminal en comparación con la escisión mesorrectal total primaria, especialmente en lesiones distales. La escisión mesorrectal total transanal puede facilitar las anastomosis más bajas, lo que podría reducir las tasas de colostomía terminal.

**OBJETIVO:**

Comparar la tasa de colostomía terminal y los resultados oncológicos de la escisión mesorrectal total transanal primaria con la escisión local seguida de la escisión mesorrectal total transanal de complemento en pacientes con cáncer rectal cT1-2N0M0.

**DISEÑO:**

Los datos se recopilaron de forma prospectiva en seis centros holandeses con gran volumen de experiencia en la escisión mesorrectal total transanal y se analizaron retrospectivamente.

**PACIENTES:**

Todos los pacientes con cáncer rectal cT1-2N0M0 que se sometieron a una escisión mesorrectal total transanal primaria o a una escisión local seguida de una escisión mesorrectal total transanal de complemento entre 2012 y 2022.

**PRINCIPALES MEDIDAS DE RESULTADO:**

El resultado principal fue la tasa de colostomía terminal. Los resultados secundarios incluyeron la fuga anastomótica, la afectación del margen de resección circunferencial, la calidad del espécimen, la recurrencia local, las metástasis a distancia, la supervivencia global y la supervivencia libre de enfermedad..

**RESULTADOS:**

Se incluyó un total de 150 pacientes con una mediana de seguimiento de 32 y 23 meses para la escisión mesorrectal total transanal primaria y la escisión local seguida de una escisión mesorrectal total transanal de complemento, respectivamente. La tasa de colostomía terminal fue significativamente menor en el grupo de escisión local seguida de escisión mesorrectal total transanal de complemento (21 %) en comparación con el grupo de escisión mesorrectal total transanal primaria (42 %, p = 0.022). Se produjeron más fugas anastomóticas en el grupo de escisión local seguida de escisión mesorrectal transanal total cde complemento (33 % frente a 18 %, p = 0.064). No se observaron diferencias en la afectación del margen de resección circunferencial ni en la calidad del espécimen. Las tasas de recidiva local a dos años fueron del 4 % para la escisión mesorrectal total transanal primaria y del 3 % para la escisión local seguida de escisión mesorrectal total transanal de complemento(p = 0.343), mientras que las metástasis a distancia se produjeron en el 8 % y el 10 % (p = 0.424), respectivamente. No hubo diferencias significativas en la supervivencia global a dos años (88 % frente a 97 %, p = 0.101) ni en la supervivencia libre de enfermedad a dos años (82 % frente a 90 %, p = 0.463) entre los grupos.

**LIMITACIONES:**

El pequeño tamaño de la muestra, que impidió la corrección de las diferencias entre los grupos, y el sesgo de selección.

**CONCLUSIÓN:**

Este estudio demostró que la escisión local seguida de la escisión transanal total del mesorrecto de complemento para el cáncer rectal cT1-2N0 no aumentó la tasa de creación de colostomía terminal ni comprometió los resultados oncológicos en comparación con la escisión transanal total del mesorrecto primaria en centros con experiencia. (*Traducción—Dr. Jorge Silva Velazco*).

Screening programs for bowel cancer have induced a substantial incidence shift toward early-stage colorectal cancer in the Netherlands.^1,2^ This has generated a growing interest in organ-preserving treatment strategies that avoid the need for extensive radical resection. This demand is particularly pronounced for rectal cancer, as radical surgery (ie, total mesorectal excision [TME]) is associated with a significant decrease in quality of life due to high ostomy rates and significant morbidity. As a result, less invasive treatment options for early rectal cancer, such as endoscopic and surgical local excision (LE), have become increasingly popular.^3–5^

LE as an organ-preserving strategy is considered oncologically safe for pT1 rectal tumors lacking histopathological risk factors that increase the risk of local recurrence, such as lymphatic and/or vascular invasion, tumor budding, and poor differentiation.^6–9^ Preoperative diagnostic imaging fails to accurately differentiate between low-risk and high-risk early rectal cancer. The LE-first strategy adopts an approach in which a diagnostic and potentially curative LE is performed as a first step to obtain histopathological assessment.^10–12^ A significant proportion of these tumors have low-risk features and do not require further treatment. Conversely, if histopathological evaluation reveals a pT1 tumor with risk factors, or if the tumor is classified as pT2 or higher, (inter)national guidelines recommend completion TME (cTME) as a second step to reduce the risk of recurrence.^8,13^

A notable concern regarding cTME is its potential association with adverse clinical outcomes. Although previous studies have reported conflicting results, cTME may cause higher rates of end colostomy, poorer specimen quality, increased risk of rectal perforation, and more frequent reinterventions compared to primary TME (pTME).^14–21^ These potential drawbacks may be attributed to the presence of fibrotic scar tissue and the compromised surgical plane resulting from the prior LE.

The transanal approach to TME (TaTME) was introduced in 2010 to address the surgical challenges associated with mid and low rectal cancer. This approach is particularly beneficial for patients with complex anatomical and clinical factors, such as low-situated rectal tumors, prior neoadjuvant radiotherapy, obesity, enlarged prostate, and men with a narrow pelvis.^22^ TaTME provides direct visualization of the tumor or scar tissue within the lumen, allowing for a precise proctectomy and optimization of the length of the rectal stump while ensuring a safe distal margin. This approach may contribute to the formation of a more efficient (low) anastomosis, with a potentially lower end colostomy rate, better specimen quality, and reduced postoperative morbidity.^23^

Literature addressing the safety of LE before TaTME as a first step in the treatment of early rectal cancer is scarce. With the increasing adoption of the LE-first strategy in the management of rectal cancer, it is important to investigate whether the attempt to preserve the rectum compromises the outcomes of TaTME surgery. The aim of this study is to compare clinical outcomes, including end colostomy rates and oncological outcomes, between primary TaTME (pTaTME) and LE followed by completion TaTME (cTaTME) in patients with cT1-2N0M0 rectal cancer.

## METHODS

### Data Collection

Patient data for this multicenter cohort study were prospectively collected from 6 high-volume centers in the Netherlands as part of an external audit evaluating the implementation of TaTME and retrospectively analyzed. All centers adhered to a nationally structured training program, including on-site proctoring and cadaver training specifically designed for postgraduate colorectal surgeons. This program was implemented at centers performing 20 or more TME procedures per year and with proficiency in laparoscopic TME.^24^

### Patient Selection

This study included all patients (aged 18 years or older) with MRI-defined cT1-2 rectal cancer, without evidence of locoregional lymph node involvement or distant metastases, who underwent either pTaTME or LE followed by cTaTME between 2012 and 2022. The surgical approach (LE first or pTaTME) was determined by the treating physician based on clinical assessment and shared decision-making with the patient. Patients who received delayed surgery for recurrence after prior LE (salvage surgery) were not included. Other exclusion criteria were cT3-4 rectal cancer, neoadjuvant treatment, suspicious mesorectal lymph nodes, and distant metastases.

### Follow-up Protocol

Although the surveillance protocol after TME evolved during the 10-year study period, it consistently included regular laboratory testing (eg, CEA), imaging (eg, CT, MRI), and endoscopic evaluation (eg, colonoscopy, sigmoidoscopy). Additional imaging and/or endoscopic evaluation was performed when an anastomotic leak was suspected. If patients were referred to other hospitals for follow-up, their data were requested. Patients were considered lost to follow-up if all attempts to obtain follow-up data (from the general practitioner or directly from the patient) were unsuccessful.

### Outcome Measures

The primary outcome of interest was the end colostomy rate at the end of follow-up. Secondary outcomes included perioperative complications such as anastomotic leakage, involvement of the circumferential resection margin (CRM), specimen quality, 2-year local recurrence rate, 2-year distant metastases rate, 2-year overall survival (OS), and 2-year disease-free survival (DFS). Intraoperative complications were defined as any adverse event occurring during surgery that required additional intervention, prolonged the procedure, or posed a potential risk to the patient’s well-being and was considered a complication in the judgment of the surgeon. Postoperative complications were classified according to the Clavien-Dindo (CD) scale, with minor morbidity defined as CD grade 2 or less and major morbidity as CD grade 3 or more. Anastomotic leakage was categorized according to the intervention required: grade A requiring no active intervention, grade B requiring active radiological or endoscopic intervention, and grade C requiring surgical reintervention. A negative CRM was defined as tumor tissue ≥1 mm from the resection margin. Specimen quality was classified as complete, nearly complete, and incomplete according to the MERCURY (Magnetic Resonance Imaging and Rectal Cancer European Equivalence Study) criteria.^25^

### Statistical Analysis

Baseline characteristics, perioperative data, and oncological outcomes were described using descriptive statistics. Categorical data were reported as frequencies and percentages and compared between groups using Pearson’s χ^2^ test or Fisher exact test. Depending on their distribution, continuous variables were either presented as mean with SD or as median with interquartile range (IQR) and compared with an unpaired *t* test or Mann-Whitney *U* test as appropriate.

Risk of recurrence, as well as OS and DFS, was estimated using the Kaplan-Meier method and compared between groups using the log-rank test. Median follow-up was estimated using the reverse Kaplan-Meier method. A 2-tailed *p* value of <0.05 was considered statistically significant. SPSS version 28 (IBM Corp, Armonk, NY) was used for all statistical analyses.

## RESULTS

**FIGURE 1.**
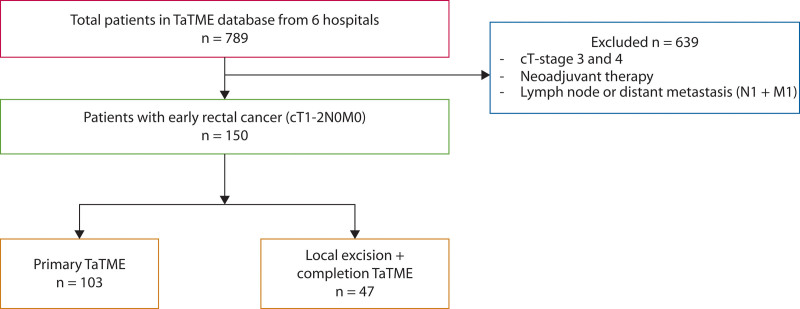
Flow chart of patient selection. TaTME = transanal total mesorectal excision.

A total of 789 patients underwent TaTME surgery between 2012 and 2022. After excluding patients with cT3-4 rectal cancer or metastases and those who received neoadjuvant treatment, a total of 150 patients were included in this study. Of these, 103 patients received pTaTME and 47 patients were treated with LE+cTaTME. The patient selection process is illustrated in Figure 1.

### Patient and Tumor Characteristics

Patient and tumor characteristics are presented in Table 1. The median follow-up was 32 (IQR, 15–47) and 23 (IQR, 10–46) months for pTaTME and LE+cTaTME , respectively. Mean BMI was significantly higher in the pTaTME group compared to the LE+cTaTME group (27 vs 26, *p* = 0.032). In addition, the pTaTME group included more patients with a cT2 tumor (92% vs 77%, *p* = 0.008). The most commonly performed LE technique was transanal endoscopic microsurgery or transanal minimally invasive surgery. Radical LE was achieved in nearly 85% of patients, of whom 20% had resection margins <1 mm.

**TABLE 1.** Baseline characteristics

*Characteristic*	*pTaTME* *(n = 103*)	*LE+ cTaTME* *(n = 47*)	*p*
Sex male	75 (72.8)	32 (68.1)	0.552
Age, mean (SD)	67.2 (9.6)	66.0 (7.4)	0.381
BMI, mean (SD)	27.1 (4.1)	25.6 (3.4)	0.032
ASA score			0.069
1	11 (10.7)	10 (21.3)	
2	74 (71.8)	25 (53.2)	
3	18 (17.5)	12 (25.5)	
cT stage			0.008
cT1	8 (7.8)	11 (23.4)	
cT2	95 (92.2)	36 (76.6)	
Distance to anorectal junction, cm, median (IQR)	3 (1–5)	2 (1–4.8)	0.289
Type of local excision	NA		NA
TEM/TAMIS		37 (78.7)	
EID		7 (14.9)	
ESD		2 (4.3)	
Polypectomy		1 (2.1)	
Plane of local excision	NA		NA
Full thickness		25 (67.6)	
Intramuscular		4 (10.8)	
Submucosal		8 (21.6)	
Missing		10	
Radicality of local excision	NA		NA
R0		24 (63.2)	
R1 (<1 mm)		8 (21.1)	
R1 (tumor in resection plane)		6 (15.8)	
Missing		9	
Time between local excision and completion surgery, weeks, median (IQR)	NA	9 (7–12)	NA

Data reported as n (%) unless otherwise indicated. ASA = American Society of Anesthesiologists; BMI = body mass index; cTaTME = completion transanal total mesorectal excision; EID = endoscopic intermuscular dissection; ESD = endoscopic submucosal dissection; IQR = interquartile range; LE = local excision; NA = not applicable; pTaTME = primarytransanal total mesorectal excision; TAMIS = transanal minimally invasive surgery; TEM = transanal endoscopic microsurgery.

### Intraoperative Outcomes

Intraoperative outcomes are shown in Table 2. The percentage of nonrestorative resections was higher in the pTaTME group compared to the LE+cTaTME group, although this difference was not statistically significant (29% vs 17%, *p* = 0.114). Despite the difference in operative time (228 minutes for pTaTME vs 261 minutes for LE+cTaTME, *p* = 0.025), no statistically significant differences were observed between the 2 groups in terms of intraoperative complications, conversion rate, ileostomy diversion, or blood loss.

**TABLE 2.** Intraoperative outcomes

*Outcome*	*pTaTME* *(n = 103*)	*LE+cTaTME* *(n = 47*)	*p*
Type of TaTME			0.182
LAR^a^	83 (81.4)	40 (85.1)	
ISR	17 (16.7)	6 (12.8)	
APR	0 (0)	1 (2.1)	
Proctocolectomy–IPAA	2 (2.0)	0 (0)	
Missing	1	0	
Conversion to laparotomy			>0.99
Yes	2 (1.9)	1 (2.1)	
No	101 (98.1)	46 (97.9)	
Restorative resection			0.114
Restorative (anastomosis)	73 (70.9)	39 (83.0)	
Nonrestorative (end ostomy)	30 (29.1)	8 (17.0)^b^	
Diverting ileostomy^c^			0.606
Yes	43 (58.9)	21 (53.8)	
No	30 (41.1)	18 (46.2)	
Intraoperative complications	21 (20.4)	12 (26.1)	0.439
Operative time, min, mean (SD)	227.8 (79.7)	260.6 (82.7)	0.025
Blood loss, mL, median (IQR)	87.5 (30–143.8)	100 (40–225)	0.347

Data presented as n (%) unless otherwise indicated. APR = abdominoperineal resection; cTaTME = completion transanal total mesorectal excision; IQR = interquartile range; ISR = intersphincteric resection; LAR = low anterior resection; LE = local excision; pTaTME = primary transanal total mesorectal excision.

a Including Hartmann procedures.

b Including 1 end ileostomy.

c Restorative procedures.

### Postoperative Morbidity

Table 3 represents the postoperative outcomes. The incidence of minor and major morbidity was similar in both groups, with minor morbidity occurring in 26% versus 21% and major morbidity occurring in 23% versus 38% in the pTaTME group and LE+cTME group, respectively (*p* = 0.165). Although not statistically significant, anastomotic leakage appeared to be more prevalent after LE+cTaTME compared to pTaTME (33% vs 18%, *p* = 0.064).

**TABLE 3.** Postoperative morbidity

*Outcome*	*pTaTME* *(n = 103*)	*LE+cTaTME* *(n = 47*)^a^	*p*
Postoperative morbidity			0.165
No	52 (50.5)	19 (40.4)	
Minor morbidity	27 (26.2)	10 (21.3)	
Major morbidity	24 (23.3)	18 (38.3)	
Anastomotic leakage^b^			0.064
Yes	13 (17.8)	13 (33.3)	
No	60 (82.2)	26 (66.7)	
Grade anastomotic leakage			0.790
A	0	0	
B	4	5	
C	8	8	
Missing	1	0	
Readmission			0.322
Yes	20 (20.8)	12 (28.6)	
No	76 (79.2)	30 (71.4)	
Missing	7	5	
Reoperation			0.171
Yes	28 (27.2)	18 (38.3)	
No	75 (72.8)	29 (61.7)	

Data presented as n (%). cTaTME = completion transanal total mesorectal excision; LE = local excision; pTaTME = primary transanal total mesorectal excision.

a Morbidity after cTaTME.

b Restorative procedures.

### Ostomy Rates

Of the patients who underwent a restorative procedure, 23% required secondary diversion due to postoperative complications in both groups (*p* = 0.206), as detailed in Table 4. At the end of follow-up, 42% of patients in the pTaTME group had an end colostomy compared to 21% in the LE+cTaTME group (*p* = 0.022).

**TABLE 4.** Ostomy rates

*Outcome*	*pTaTME* *(n = 103*)	*LE+cTaTME* *(n = 47*)	*p*
Secondary diversion^a^	17 (23.3)	9 (23.1)	0.206
Ileostomy	7 (9.6)	7 (17.9)	
Anastomotic leakage	7	7	
End colostomy	10 (13.7)^b^	2 (5.1)	
Anastomotic leakage	6	2	
Perianal abscess	1	0	
Rectovaginal fistula	1	0	
Recurrence treatment	1	0	
Unknown	1	0	
No	56 (76.7)	30 (76.9)	
Ostomy reversed			0.067
Ileostomy	32 (41.6)	20 (60.6)	
End colostomy	0	0	
No	45 (58.4)	13 (39.4)	
Missing	7	5	
Ileostomy at end of follow-up			0.345
Yes	5 (5.2)	4 (9.5)^c^	
No	91 (94.8)	38 (90.4)	
Missing	7	5	
End colostomy at end of follow-up			0.022
Yes	40 (41.7)	9 (21.4)	
No	56 (58.3)	33 (78.6)	
Missing	7	5	

Data presented as n (%). cTaTME = completion transanal total mesorectal excision; LE = local excision; pTaTME = primary transanal total mesorectal excision.

a Restorative procedures.

b Six patients underwent conversion from ileostomy to colostomy.

c Including 1 end ileostomy.

### Histopathological Outcomes

Histopathological outcomes are presented in Table 5. Both groups had comparable histopathological outcomes, including similar rates of specimen quality (complete in 85% of pTaTME cases vs 77% of LE+cTaTME cases, *p* = 0.399) and negative CRM (97% for pTaTME vs 96% for LE+cTaTME, *p* = 0.649).

**TABLE 5.** Histopathological outcomes

*Outcome*	*pTaTME* *(n = 103*)	*LE+cTaTME* *(n = 47*)^a^	*p*
pT stage			0.064
pT0	4 (3.9)	0	
pT1	15 (14.6)	15 (31.9)	
pT2	68 (66.0)	25 (53.2)	
pT3	16 (15.5)	7 (14.9)	
pN stage			0.474
pN0	81 (78.6)	40 (87.0)	
pN1	20 (19.4)	6 (13.0)	
pN2	2 (1.9)	0 (0)	
Missing	0	1	
Specimen quality			0.399
Complete	88 (85.4)	36 (76.6)	
Nearly complete	8 (7.8)	6 (12.8)	
Incomplete	7 (6.8)	5 (10.6)	
CRM involvement			0.649
Yes	3 (2.9)	2 (4.3)	
No	100 (97.1)	45 (95.7)	
Lymph nodes removed, mean (SD)	18.2 (8.7)	14.7 (6.1)	0.013

Data presented as n (%) unless otherwise noted. CRM = circumferential resection margin; cTaTME = completion transanal total mesorectal excision; LE = local excision; pTaTME = primary transanal total mesorectal excision.

a Histopathological assessment of completion TaTME specimen.

### Oncological Outcomes

**FIGURE 2.**
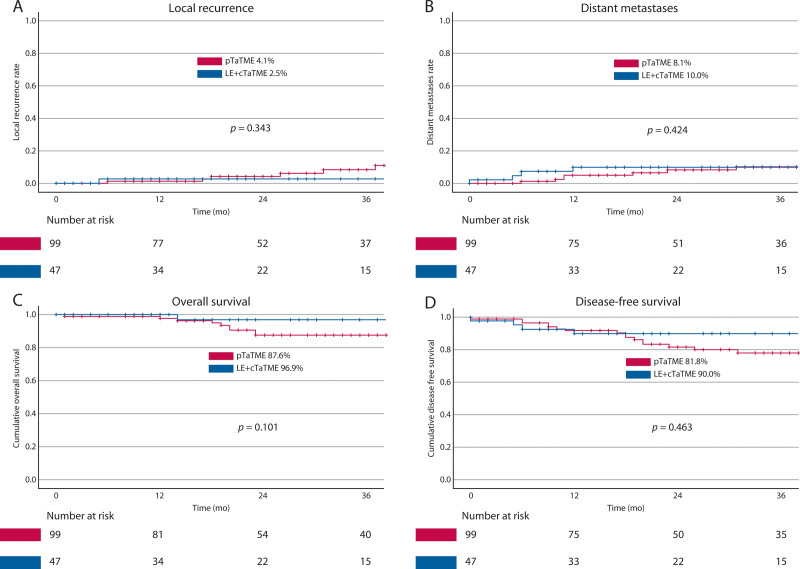
Two-year recurrence and survival outcomes. A, Two-year local recurrence rate for pTaTME (4.1%) vs LE+cTaTME (2.5%, *p* = 0.343). B, Two-year distant metastases rate for pTaTME (8.1%) vs LE+cTaTME (10.0%, *p* = 0.424). C, Two-year overall survival for pTaTME (87.6%) vs LE+cTaTME (96.6%, *p* = 0.101). D, Two-year disease-free survival for pTaTME (81.8%) vs LE+cTaTME (90.0%, *p* = 0.463). cTaTME = completion transanal total mesorectal excision; LE = local excision; pTaTME = primary transanal total mesorectal excision.

The oncological outcomes are summarized in the Kaplan-Meier curves in Figure 2. Two-year local recurrence rates were 4% in the pTaTME group and 3% in the LE+cTaTME group (*p* = 0.343). The 2-year distant metastases rate was comparable between the 2 treatment groups, with 8% in the pTaTME group versus 10% in the LE+cTaTME group (*p* = 0.424).

Survival data are also illustrated in Figure 2. The 2-year OS rates were 88% and 97% for pTaTME and LE+cTaTME, respectively. The 2-year DFS rates were 82% for pTaTME and 90% for LE+cTaTME. Log-rank tests showed no statistically significant difference in OS (*p* = 0.101) or DFS (*p* = 0.463) between the 2 groups.

## DISCUSSION

This large prospective cohort study comparing pTaTME with LE+cTaTME demonstrated that the LE-first strategy for cT1-2N0 rectal cancer did not increase the end colostomy rate, nor compromise oncological outcomes in experienced centers. These findings suggest that LE as an initial step is a safe and effective treatment strategy for managing early rectal cancer.

Previous literature, including 2 nationwide studies from the Netherlands and Norway comparing laparoscopic pTME with cTME, demonstrated a significantly higher end ostomy rate after completion surgery.^2,26,27^ Although we hypothesized that cTaTME would yield similar end colostomy rates compared to pTaTME due to the advantages of the transanal approach, the significantly lower incidence observed in the cTaTME group was unexpected. As the LE-first strategy is unlikely to account for the observed difference, possible explanations include the surgeons’ expertise in performing completion surgery in the present study. In addition, patients who initially underwent LE may have had a greater preference for anastomotic preservation, potentially leading to increased effort to avoid a permanent colostomy. Finally, the decision to proceed with a primary anastomosis versus a nonrestorative approach is influenced by a variety of clinical factors, including BMI, patient comorbidities, tumor stage, tumor height, surgeon preference, institutional practice, intraoperative findings, or a combination thereof. However, the limited sample size of this study precluded regression analysis to adjust for these potentially confounding factors. Although recent data on cTME using transanal techniques remains limited, a study describing a case-matched cohort (n = 87) including both TaTME and robotic TME found no difference in the proportion of patients with an end ostomy between primary and completion surgery.^28^ A study specifically focusing on cTaTME (n = 25) reported a higher short-term end ostomy rate than that observed in the present study (36% vs 21%).^29^ This difference may be due to more advanced tumor stages, administration of neoadjuvant radiotherapy, and less experience with TaTME at the participating centers compared to the present study.^29^

Consistent with previous literature, this study demonstrated comparable postoperative morbidity between pTaTME and LE+cTaTME.^15,17–21,28,30,31^ Although there was a trend toward a higher incidence of anastomotic leakage after LE+cTaTME, this did not translate into more anastomotic take-down (ie, end colostomy rate) compared to pTaTME. This trend was also reported by a recent study showing an anastomotic leak rate of 26.3% for cTME versus 10.7% for pTME without reaching statistical significance.^28^ Koedam et al^29^ reported a remarkably lower incidence of anastomotic leakage (4%) and a lower percentage of major morbidity (20%) after cTaTME compared to this cohort (33% and 38%, respectively). These results may have been influenced by several factors, including the proportion of low rectal tumors and male patients with a high mean BMI in our cohort, both of which are known to correlate with increased anastomotic leak rates.^32^ Although the potentially increased risk of leakage after cTaTME in this study did not result in a significantly higher percentage of end colostomies, it should be carefully discussed with patients during shared decision-making.

The CRM involvement rates observed in the present study are consistent with previous literature, showing no significant differences between pTME and cTME.^28–30,33–35^ However, a meta-analysis by Wyatt et al^30^ comparing pTME with cTME reported a significantly higher percentage of incomplete specimens after completion surgery (risk ratio, 3.06; 95% CI, 1.41–6.62; *p* = 0.005). This finding may be attributed to the inclusion of patients treated with neoadjuvant therapy, which causes scarring and fibrosis in the mesorectal plane, complicating surgical dissection.^30^ Incomplete resection is a clinical concern, as it is associated with worse oncological outcomes.^36,37^ The absence of a significant difference in specimen quality between primary and completion surgery in this study could be attributed to the use of the transanal technique, as previous studies comparing laparoscopic pTME with pTaTME also demonstrated superior specimen quality with TaTME.^35^ In addition, cTaTME has been associated with low R1 rates, which may be due to the improved visualization within the lumen, allowing for more precise distal resection.^29,35^

Although the pTaTME group included a greater proportion of patients with more advanced tumor stages, this did not result in a statistically significant difference in oncological outcomes between the 2 treatment groups. Although data on cTaTME are not yet available, studies comparing pTME and cTME have reported similar oncological results. A meta-analysis found no differences in local or distant recurrence after a minimum follow-up of 25 months.^30^ In addition, Burghgraef et al^28^ showed no differences in the 3-year local recurrence rate (3.4% for cTME at 30 months vs 8.6% for pTME (*p* = 0.43) and the 3-year distant metastases rate (3.4% for cTME vs 12.1% for pTME, *p* = 0.25). This study also showed a 3-year OS of 93.1% for cTME versus 94.8% for pTME (*p* = 0.71) and a 3-year DFS of 89.7% for cTME versus 81.0% for pTME (*p* = 0.43), which are consistent with our findings.^28^ Although the OS for pTaTME in our cohort was lower than reported in the literature, the small sample size precludes definitive conclusions.

Given the relatively high proportion of surgically challenging patients included in this study (male, obese patients with tumors located relatively low in the rectum), the generalizability of these findings to the broader population with early rectal cancer may be limited. However, the LE-first strategy using the transanal technique appears to be safe in patients with challenging characteristics, without increasing the end colostomy rate or compromising oncological safety. The advantage of this approach is even greater when considering that a substantial proportion of patients who undergo full-thickness LE for low-risk tumors can be managed with surveillance alone, avoiding surgery-related morbidity.

Despite a cN0 classification, 13% of the patients in the LE+cTaTME group had positive lymph nodes (pN+), highlighting the importance of completion surgery in the presence of risk factors on histopathological assessment of the LE specimen. Due to the relatively high morbidity associated with TME surgery, less invasive treatment options after LE, such as adjuvant chemoradiotherapy, are currently being investigated.^38^

The present study has several limitations. The relatively small patient cohort, consisting only of patients from high-volume centers with TaTME expertise, may limit the generalizability of the findings. In addition, the nonrandomized treatment allocation and the inclusion of patients specifically referred for restorative procedures may have introduced selection bias. Furthermore, although statistically significant differences in BMI and T-stage were observed between the 2 treatment groups, the small sample size precluded robust multivariate analysis to adjust for these potentially confounding factors. Nevertheless, this study represents the largest prospective cohort on cTaTME to date.

## CONCLUSION

This study demonstrated that LE followed by cTaTME for cT1-2N0 rectal cancer neither increased the end colostomy rate nor compromised oncological outcomes when compared to pTaTME at centers experienced in TaTME. These findings suggest that the LE-first strategy is a safe and effective treatment option for patients with early rectal cancer.

## ACKNOWLEDGMENTS

This study was approved by the Medical Ethics Committee of the Amsterdam University Medical Centre and deemed exempt from the Dutch Medical Research Involving Human Subjects Act. The study was conducted and reported in accordance with the Strengthening the Reporting of Observational Studies in Epidemiology (STROBE) statement.
